# Prevalence of exclusive breastfeeding among mothers in the informal sector, Kampala Uganda

**DOI:** 10.1371/journal.pone.0239062

**Published:** 2020-09-24

**Authors:** Phoebe Nabunya, Ruth Mubeezi, Phyllis Awor

**Affiliations:** 1 Department of Biostatistics and Epidemiology, Makerere School of Public Health, Kampala, Uganda; 2 Department of Disease Control and Environmental Health, Makerere School of Public Health, Kampala, Uganda; 3 Department of Community Health and Behavioral Sciences, Makerere School of Public Health, Kampala, Uganda; University of North Carolina at Greensboro, UNITED STATES

## Abstract

Exclusive breastfeeding (EBF) for the first six months of life is effective in preventing infant morbidity and mortality. However, 36% of Ugandan children below 6 months are not breastfed exclusively despite its active promotion. This study determined the prevalence and factors associated with exclusive breastfeeding among mothers working in the informal sector in Kampala district. A community based cross-sectional study targeting 428 interviews with mothers with children aged 0–5 months was conducted. Analysis was done using modified Poisson regression in Stata version 14. The prevalence of exclusive breastfeeding was 42.8%. The factors associated with exclusive breastfeeding included: attending antenatal care at least 4 times (APR = 1.24; 95% CI: 1.01–1.51), intention to exclusively breastfeed for 6 months (APR = 1.26; 95% CI: 1.01–1.57) or longer (APR = 1.38; 95% CI: 1.06–1.76), proper breastfeeding practices (APR = 4.12; 95% CI: 2.88–5.90), age of the infant (APR = 0.78; 95% CI: 0.65–0.94) and (APR = 0.48; 95% CI: 0.39–0.60) for children aged 2–3 and 4–5 months respectively and working in a lower position (APR = 0.68; 95% CI: 0.55–0.83). Mothers should be encouraged to attend antenatal care where they learn about the benefits of exclusive breastfeeding to children below 6 months hence enabling them to make informed decisions about exclusive breastfeeding. The government of Uganda should ensure maternity leave benefits of the employment act are enforced in the informal sector to allow women to practice EBF.

## Introduction

Exclusive breastfeeding (EBF) is defined as feeding infants only breast milk, either directly from the breast or expressed, with no addition of any liquid or solids apart from drops or syrups consisting of vitamins, mineral supplements or medicine, and nothing else [1]. Although over 90% of children in Uganda are breastfed at some point, the percentage of children exclusively breastfed decreases sharply with age from 83% in infants 0–1 month to 69% among those 2–3 months and further to 43% among infants aged 4–5 months [2].

Globally several factors have been found to be associated with infant feeding practices. These include income, education, wealth and employment [3–6]. Work has also been found to hinder EBF as mothers stop breastfeeding soon after returning to work especially when the environment at work is not conducive for continued breastfeeding. The lack of EBF has in turn been associated with increasing child malnutrition [5, 6].

For a work place to support breastfeeding the employer should support it. However, several studies like the one done in the United States of America (USA) found that employers generally have a negative attitude towards breastfeeding as it takes time off work making women less efficient [5]. Other authors recommended that the work place should have a private space with a locking door, access to clean safe water, time to express milk at work, education and professional breastfeeding support, and adequate refrigeration in order to be supportive [7, 8].

In Uganda, the employment act of 2006 states that women are entitled to a 60 day paid leave from their employer following child birth or miscarriage [9]. In addition to this, the parliament is also advocating for making work places breastfeeding friendly through establishing breastfeeding rooms and allowing breastfeeding breaks for breastfeeding mothers [10]. Even though, openly accessible to both employees and employers, this act is emphasized for the formal and hardly in the informal sector. In addition to this gap, the recommended facilities to support EBF are almost impossible to install in the informal workplaces due to lack of resources [11].

The Informal sector is largely made up of micro-firms who are below the minimum threshold for small business income tax and it employs 71% of women in Uganda [11, 12]. These include markets, shops (retail, hardware, wholesale, and kiosks), agriculture, small scale saloons and restaurants. An infant feeding survey in the UK found that 90% mothers in managerial and professional occupations practiced EBF compared to only 61% in routine and manual occupations [13]. However, study reported that in Asia, maternal informal employment was associated with higher odds of exclusive breastfeeding compared to non-employment [14]. Even though several studies on EBF have been conducted in Uganda, little is known about the situation on EBF in Uganda’s informal sector. The purpose of this study was to determine the prevalence and associated factors of exclusive breastfeeding among mothers working in the informal sector in Kampala in order to inform the breastfeeding guidance and support to the mothers especially in the context of informal sector.

## Methodology

The study was conducted in Kampala district where 51.9% of the working population is women [15]. The respondents were mothers with children 0–5 months working in the main informal sector jobs in Kampala district namely general shops, food shops/restaurants, furniture shops, small scale salons and markets [16].

Sample size was calculated using the Bennet formula for cluster surveys; C = *Z*^2^*P*(1−*P*)*D*/*d*^2^*b* [17]. The sample size was based on an EBF prevalence among working women from a recent study in neighboring Tanzania which was 20% [18], confidence interval of 95% (at a Z-score of 1.96), absolute error of precision of 5%, a design effect of two [2] and a non-response rate of 13% [19]. This gave us 20 clusters and a sample size of 428; with a minimum of 22 respondents from 8 clusters and 21 from the rest.

Out of the five divisions in Kampala, two (Kawempe and Rubaga) were selected by ballot. A complete list of the parishes and villages in each division was obtained from the division offices and two parishes from each randomly selected followed by random selection of five villages from each parish. The center of the village was identified and by tossing a coin to decide the direction, the first household in that direction was identified. The next households were selected consecutively. Only one mother-child pair was taken from each household and the youngest child in each household was taken as the index child. If the mother had twins, the index child was the younger twin. This process continued until the target mother-child pairs had been interviewed in each village. Households where the data collectors did not find a woman working in the informal sector with a child in this age group were skipped until a household which meets the inclusion criteria was found. For Makerere III parish, enumeration continued into 5 other villages as we failed to meet the target mother-child pairs in each village. (This was probably due to the high number of hostels inhabited by students). A summary of the selection process is presented in Table 1.

**Table 1:** Selection of the respondents.

**Selected Divisions**	Number of Parishes	Selected parishes	Number of villages /parishes	Number of visited villages per parish	Number of women targeted	Number of women interviewed	Response rate (%)
Rubaga	13	Busega	14	5	110	110	100
		Kasubi	26	5	108	105	97
Kawempe	19	Makerere III	43	10	105	108	103
	2	Mulago2	27	5	105	105	100

The dependent variable was exclusive breastfeeding measured as having received only breast milk in the previous 24 hours. The independent variables included infant’s age in months, infant’s sex, maternal age measured in completed years, marital status, parity, inter delivery interval, intention of exclusively breastfeed measured as duration of planned EBF before delivery, mode of delivery, maternal education, education of spouse, spouse stayed at home after delivery and assisted in feeding the child with expressed breast milk, occupation, position at work, received leave time after delivery measured as days/months before returning to work, duration of leave time, taking child to work, presence of breastfeeding facilities at work, breastfeeding frequency, maternal knowledge with a mother being considered knowledgeable if she answered three of the four following questions correctly:1) proper initiation time ii) feeds offered to children below 6 months iii) knowledge of at least one benefit of EBF iii) recommended breastfeeding frequency, and iv) recommended duration of EBF. Others included receipt of breastfeeding counseling and education, attitude; with a mother being considered to have a positive attitude if she answered all the following statements positively: i) It is good to exclusively breastfeed your baby for six months ii) it is not difficult to exclusively breastfeed my baby for six months, iii) I feel confident when breastfeeding my child, iv) I can confidently express breast milk for my child, number of antenatal visits, and practices at delivery (rooming in, skin to skin contact, time (hours) of initiation, pre-lacteals, and professional support);with those who did 3 out of the 5 considered to have good practices. This study was conducted with ethical approval from the Higher Degrees, Research and Ethics Committee (HDREC) at School of Public Health, Makerere University (MakSPH). Written informed consent was collected from all subjects. Participants were informed that their participation was voluntary and they could stop the interviews at any time.

The data collection was done between March and June 2018. Research assistants underwent a two-day training to ensure that they understood the study objectives and familiar with the tools and equipped with basic ethical principles skills when dealing with human subjects. A semi-structured questionnaire was designed by adopting questions on EBF from a tool on guidelines for assessing nutrition-related knowledge, attitudes and practices by the Food and Agricultural Organisation (FAO) and Infant and child feeding indicators measurement guide by Food And Technical Assistance (FANTA) [20, 21]. The tool (S1 and S2 Appendices) was translated into Luganda, and pretested in one of the villages in Kampala which had not been sampled for the study. This aided further refining of the tool translations to phrases that elicited the desired response. The Principal Investigator reviewed the data collected for completeness and accuracy daily throughout the study period.

The data was analysed in Stata version 14.0. Descriptive statistics like the mean with their standard deviation, medians with their interquartile ranges (IQR) and proportions were used to present the participants’ characteristics, and their EBF practices. The proportion of mothers practicing exclusive breastfeeding was computed. In this study, the prevalence of EBF was not rare (>10%). Therefore, we used a modified Poisson regression with robust variances to identify factors associated with exclusive breastfeeding. Prevalence ratios (PR) were the preferred measure of association over odds ratios because the latter would overestimate the strength of the association [22]. Associations were presented with 95% confidence intervals (CI).

The factors with a P-value <0.2 at bivariate analysis were included in the multivariate analysis to obtain adjusted prevalence ratios. Model building was done by stepwise elimination. A model with Pearson chi-square >0.05 and likelihood ratio closest to zero was considered a good fit.

## Results

The infants involved in the study comprised 211/428 (49.3%) males and 217/428 (50.7%) females, majority of whom were second and third born children (155/428; 36.2%) with a birth interval of two or more years (198/281; 70.2%). The infants had a mean age of 3.5±1.56 months. Majority (335, 78.3%) of the mothers lived in male headed households who were mainly their spouses (313/427; 73.6%) and had attained secondary education (264/427; 61.8%). More descriptive factors are presented in Table 2.

**Table 2:** Socio-demographic and household characteristics of study participants (N = 428).

Factor	EBF N (%), n = 183	No EBF N (%), n = 245	Total N (%); N = 428
**Sex of the child**	**n = 183**	**n = 245**	**N = 428**
Male	82(44.8)	129(52.7)	211(49.3)
Female	101(55.2)	116(47.3)	217(50.7)
**Age of infant in months**	**n = 183**	**n = 245**	**N = 428**
0–1	52(28.4)	8(3.3)	60(14.0)
2–3	61(33.3)	49(20.0)	110(25.7)
4–5	70(38.3)	188(76.7)	258(60.3)
**Age of mothers in years**	**n = 183**	**n = 245**	**N = 428**
15–19	15(8.2)	19(7.8)	34(7.9)
20–24	54(29.5)	79(32.2)	133(31.1)
25–29	63 (34.4)	71(29.0)	134(31.3)
30–34	29(15.8)	45(18.3)	74(17.3)
35+	22(12.0)	31(12.7)	53(12.4)
**Education of mother**	**n = 183**	**n = 245**	**N = 428**
None	7(3.8)	11(4.5)	18(4.2)
Primary and below	63(34.4)	83(33.8)	146(34.1)
Secondary	90(49.2)	124(50.6)	214(50.0)
Tertiary	23(12.6)	27(11.0)	50(11.7)
**Birth order**	**n = 183**	**n = 245**	**N = 428**
1	63(34.4)	83(33.9)	146(34.1)
2–3	73(39.9)	8233.5)	155(36.2)
4–6	42(22.9)	69(28.2)	111(25.6)
7+	5(2.7)	11(4.5)	16(3.7)
**Previous birth interval**	**n = 119**	**n = 163**	**N = 281**^a^
<2 years	32(26.9)	52(31.9)	84(29.8)
≥2 years	87(73.1)	111(68.1)	198(70.2)
**Occupation**	**n = 183**	**n = 245**	**N = 428**
Market vendors	37(20.2)	51(20.8)	88(20.6)
Shop attendants	50(27.3)	76(31.0)	126(29.4)
Saloons	28(15.3)	24(9.8)	52(12.2)
Restaurants/ bars	50(27.3)	72(29.4)	122(28.5)
Agriculture	18(9.8)	22(9.0)	40(9.4)
**Marital status**	**n = 183**	**n = 245**	**N = 428**
Married	148(80.9)	182(74.3)	330(77.1)
Single/divorced/widowed	35(19.1)	63(25.7)	98(22.9)
**Position at work**	**n = 183**	**n = 245**	**N = 428**
Owner	83(45.4)	84(34.3)	167(39.0)
Managerial	37(20.2)	23(9.4)	60(14.0)
Other	63(34.4)	138(56.3)	201(46.0)
**Spousal Support in breastfeeding**	**n = 145**	**N = 177**	**N = 322^b^**
Yes	23(15.9)	32(18.1)	55(17.1)
None	122(84.1)	145(81.9)	267(82.9)
**Leave time after delivery**	**n = 183**	**N = 245**	**N = 428**
Got	161(88.0)	211(86.1)	372(86.9)
Did not get any	22(12.0)	34(13.9)	56(13.1)
**Duration of leave time**	**n = 109**	**n = 152**	**N = 261**
<3 months	60(55.0)	71(46.7)	131(50.2)
≥3 months	49(45.0)	81(53.3)	130(49.8)
**Time spent at work each day**	**n = 182**	**n = 245**	**N = 427**
<8 hours	52(28.6)	69(28.2)	121(28.3)
8 hours	7(3.8)	14(5.7)	21(4.9)
>8 hours	123(67.6)	162(66.1)	285(74.6)
**Takes child to work**	**n = 183**	**n = 245**	**N = 428**
Yes	91(49.7)	123(50.2)	214(71.8)
Sometimes	72(39.3)	58(23.7)	130(30.4)
No	20(10.9)	64(26.1)	84(28.2)
**Breastfeeding Facilities at work**	**n = 178**	**n = 244**	**N = 422**
None	141(79.2)	208(85.2)	349(82.7)
A private corner/room for baby	30(16.9)	28(11.5)	58(13.7)
Area to store expressed breast milk	7(3.9)	8(3.3)	15(3.6)
**Intention to exclusively breastfeed**	**n = 183**	**n = 245**	**(N = 428)**
<6 months	49(26.8)	129(52.7)	178(42.9)
6 months	98(53.6)	91(37.1)	189(45.5)
>6 months	30(16.4)	18(7.3)	48(11.6)
Had no plans in mind	6(3.3)	7(2.9)	13(3.0)
**Breastfeeding practices at delivery**	**n = 183**	**N = 245**	**(N = 428)**
Poor	29(15.8)	188(76.7)	217(40.0)
Proper	154(84.2)	57(23.3)	211(60.1)
**Attitude (N = 421)**	**n = 242**	**n = 197**	**n = 421**
Poor	2 (0.8)	6 (3.4)	8(1.9)
Positive	240 (99)	173 (97)	413(98)

^a^ Most mothers were having their first child

^b^ Some women did not know the details about their spouses

### Prevalence of exclusive breastfeeding

The prevalence of exclusive breastfeeding was 183/428; (42.8%). In total, 131 (30.6%) of the children received pre-lacteal feeds mainly warm water (40.5%) or glucose solution (35.5%).On analysis of the prevalence of EBF by the infants’ age, (Table 3), EBF increased slightly from 85.7% at zero months to 87.2% at one month and then rapidly dropped and to its lowest (24.6%) among five months old children. At the time of the study, none of the respondents fed their child expressed breast milk, however 76 (18%) had ever expressed breast milk for their child when critically ill in the past (Table 3).

**Table 3:** Prevalence of exclusive breastfeeding by infant’s age.

Age/months	Number of infants (N)	Proportion of children exclusively breastfed	
		Number (n)	Percentage (%)
0	21	18	86
1	39	34	87
2	53	37	70
3	57	24	42
4	91	29	32
5	169	41	25

### Factors associated with exclusive breastfeeding

The adjusted prevalence ratios (APR) in Table 4 indicate that after controlling for intention to exclusively breastfeed, position at work, taking child to work, number of times in antenatal care (ANC) and breastfeeding practices at delivery, the prevalence of EBF was 22% less among children 2–3 months (APR = 0.78; 95% CI: 0.65–0.94) and 52% less among children 4–5 months (APR = 0.48; 95% CI: 0.39–0.60) compared to children 0–1 month.

**Table 4:** Determinants of exclusive breastfeeding among mothers working in the informal sector.

Factor	UPR	CI	APR	CI
**Sex of the child**				
Male	1			
Female	1.20	0.96–1.49		
**Age of infant in months**				
0–1	1		1	
2–3	0.64	0.53–0.78***	0.78	0.65–0.94*
4–5	0.31	0.25–0.39***	0.48	0.39–0.60***
**Education of mother**				
None	1			
Primary and below	1.12	0.60–2.04		
Secondary	1.08	0.59–1.97		
Tertiary	1.18	0.62–2.27		
**Intention to exclusively breastfeed**				
<6 months	1		1	
6 months	1.89	1.43–2.48***	1.26	1.01–1.57*
>6 months	2.27	1.64–3.14***	1.38	1.06–1.76*
Had no plans in mind	1.68	0.89–3.16	1.18	0.68–2.00
**Position at work**				
Owner	1		1	
Managerial	1.24	0.96–1.60	0.90	0.73–1.11
Other	0.63	0.48–0.81***	0.68	0.55–0.83***
**Takes child to work**				
Yes	1		1	
Sometimes	0.56	0.37–0.85**	0.71	0.51–1.0
No	1.30	1.05–1.62**	1.05	0.88–1.25
**Number of times in ANC**				
<4	1		1	
>4	1.46	1.13–1.88**	1.24	1.01–1.51*
**Received counseling and education N = 428**				
Did not receive	1			
Received	1.31	1.02–1.67*		
**Breastfeeding frequency**				
<8 times	1		1	
8+ times	1.38	1.08–1.76**	4.12	2.88–5.90***

* = P<0.05

** = P<0.01

*** = P<0.001 UPPR = unadjusted Prevalence ratio, APR = Adjusted Prevalence ratio, CI = Confidence interval ANC = antenatal care

Compared to women who intended to exclusively breastfeed for less than six months, the prevalence of EBF was 26% (APR = 1.26; 95% CI: 1.01–1.57) higher for women who intended to exclusively breastfeed for at least 6 months and 38% (APR = 1.38; 95% CI: 1.06–1.76) higher for those who intended to exclusively breastfeed for longer after controlling for age of infant, position at work, taking child to work, number of times in ANC and breastfeeding practices at delivery. However, there was no difference in exclusive breastfeeding between those who had made no plans and the ones who had planned to exclusively breastfeed for less than 6 months (APR = 1.18; 95% CI: 0.68–2.00).

After controlling for intention to exclusively breastfeed, age of infant, taking child to work, number of times in ANC and breastfeeding practices at delivery, the prevalence of EBF among women who worked in lower calibers (cleaners, assistants, waitresses, sales) was 32% less compared to that among women who owned the business (APR = 0.68; 95% CI: 0.55–0.83). However, EBF among women who worked in a managerial position was not different from those who owned the businesses (APR = 0.9; 95% CI: 0.73–1.11).

While attending ANC was not a significant predictor of EBF, the prevalence of EBF was 24% higher among women who went for ANC at least 4 times compared to those who went less after controlling for the infant’s age, intention to exclusively breastfeed, position at work, taking child to work and breastfeeding practices at delivery (APR = 1.24; 95% CI: 1.01–1.51).

The prevalence of EBF was also higher among women who scored higher in the overall breastfeeding practices at delivery compared to those with a low score after controlling for age of infant, intention to exclusively breastfeed, position at work, taking child to work and number of times at ANC (APR = 4.12; 95% CI: 2.88–5.90). Table 4 has more details.

## Discussion

The prevalence of exclusive breastfeeding in this study was 42.8%. It declined from 85.7% among children aged zero months to 24.6% among children five months old. The factors associated with EBF in this study were age of the infant, position of the mother’s job at work, intention to exclusively breastfeed, attending ANC at least 4 times, and proper breastfeeding practices at delivery.

At 42.8%, the prevalence of EBF among children ≤5 months in this study is below the national prevalence of 66% and the 90% recommended by WHO demonstrating a wide gap between the desired and actual practice [23]. This is likely due to the extended time mothers had to stay away from their children for long hours while at work. In contrast to this finding, a survey conducted in Kawempe division, found a higher (56.3%) prevalence of EBF, close to the national prevalence [24]. However, this survey were not restricted to women working in the informal sector and involved stay at home mothers who may have faced fewer barriers to EBF. Similar to our findings, a study conducted among women working in pastoralism in Ghana found EBF prevalence of 58% which was lower than their national prevalence of 64%. Consistent with these findings, another study conducted in Ethiopia found the prevalence of EBF at 48%, which was also lower than the 52% at national level [25, 26].

This study found that exclusive breastfeeding reduced with increasing infant’s age. This may largely be due to the fact that mothers did not get adequate maternity leave as most got less than 3 months if any. In addition, the work places did not have a conducive environment to support mothers maintain EBF as most mothers either had no leave or did not get sufficient leave time, could not carry their children to work and spent over 8 hours separated from their children. This finding is comparable to what is reported in the Uganda Demographic Health Survey (UDHS) of 2016 where EBF dropped by half from 83% among infants 0–1 months to 43% among children 4–5 months [27]. The steep drop registered in EBF with increasing infant age registered in this study is also recorded in the study done in Kawempe division where EBF dropped by 74% between the two age groups [24]. Similarly, a study in Tanzania found that women in the informal sector returned to work soon after delivery in order to access the income needed to fulfill their responsibility of feeding their families, denying them a chance to practice EBF [28]. Furthermore, studies in Uganda and Eastern Africa showed the same trend particularly the informal sector where it was mainly attributed to lack of leave time. However, in these studies women also stated other factors including insufficient breast milk, child demanding for food, child rejecting the breast and fear of transmitting HIV to their children [28–31].

To support mothers in addressing some of these challenges, Uganda formulated breastfeeding policies such as the Baby-friendly Hospital Initiative (BFHI) intended to encourage breastfeeding through providing health education along with maternity and neonatal services [32]. Furthermore, the employment act of 2006 also provides for a 60 day paid leave for working women following child birth [9]. Even though, this period is insufficient to allow for the 6 months of EBF, most mothers reported receiving no maternal leave or receiving less than the recommended 3 months. They could also not take their babies to work forcing them to introduce other feeds. Uganda’s parliament has been advocating for improvement of work places to support breastfeeding through the creation of breastfeeding rooms, breastfeeding breaks, availing a fridge to store expressed breast milk, which have been adopted in some formal work places. However no policy revisions have been done [10].

The position of the woman at her work place determined whether she breastfed exclusively or not. Those who were owners/managers were more likely to exclusively breastfeed probably because they had more authority and were able to take breastfeeding breaks compared to those they employed. This is consistent with a survey conducted in USA which found that mothers in managerial positions practiced longer exclusive breastfeeding compared to those in routine positions [13]. Relatedly, qualitative studies conducted in USA and India found that lack of flexible work schedules, insufficient break times, and demanding work schedules were the major barriers to exclusive breastfeeding among women working in low cadre positions [33, 34]. In contrast, a study conducted in Ghana found EBF among women in the informal sector to be significantly higher compared to their counterparts in formal employment due to the flexibility of their work schedule allowing them to exclusively breastfeed without the any interference from their work [35]. A systematic review of studies conducted in low and middle income countries found that maternal employment compared to non-employment was not associated with EBF, apart from Asia where the women in informal employment were found to have higher odds of EBF largely because infants in this region accompanied their mothers to work [14].

This study also found that intention to exclusively breastfeed for six months or more was associated with exclusive breastfeeding. This is probably because mothers who planned to exclusively breastfeed for six months or more already have the structures to enable them or put measures in place that enable them to continue with EBF even when faced with challenges. Similar to these findings, a systematic review of studies carried out on modifiable factors that positively influence exclusive breastfeeding duration to 6 months postpartum reported that intention to breastfeed was an important predictor of actual breastfeeding practices including duration of EBF. Futhermore, a study from USA found that if a woman intends to breastfeed before delivery, she has a higher chance of exclusively breastfeeding and maintaining the practice even if challenged. This is also in agreement with a systematic review from studies done in multiple countires which used the theory of planned behaviour by Azjen to explain that for successful execution of a breastfeeding practices, intention was associated with the proper atitude and commitment that translated into actual action [36–39].

Similar to other studies, attending antenatal care at least 4+ times was a significant predictor of exclusive breastfeeding [27, 40]. According to the Uganda demographic survey (2016), most mothers in Uganda, especially those in urban areas get professional counseling and information on health from health facilities, with information on EBF being received mostly from ANC [27]. Similarly, a study on health seeking behavior and challenges in utilizing health facilities in Wakiso district, found that urban women seek care from health facilities and hardly from the community health workers despite their pivotal role in improving breastfeeding practices in low and middle income countries through the one-to-one reinforcement of knowledge and skills for successful breastfeeding [41, 42]. The increased knowledge and attitudinal changes on infant feeding and the nutritional values of breast milk attained from ANC may be the reason mothers who attended ANC at least 4 times practiced EBF [43]. Similar to the 60% reported in the Uganda Demographic survey in 2016, this study found that 62.1% of the mothers attended ANC at least 4 times [44]. This finding suggests that promoting universal coverage of ANC could be effective in increasing ANC among this group as some studies indicate that women who attend 4 or more ANC visits tend to be wealthier. However, wealthier women have also been found to have lower EBF rates due to barriers including work [3–6, 45, 46].

The duration of leave time was not associated with EBF in this study possibly due to the fact that majority of the mothers did not get any leave time. This is in contradiction with a study done among African American women which found that women who return to work less than 3 months after delivery were less likely to exclusively breastfeed their children compared to their counterparts who received a longer leave time [34]. Comparably, a study done in Uganda by ILO (2005) found that the maternity protections in the employment act cover mainly women in the private and public sector leaving out those who work in the family undertakings which comprises majority of the informal employment in Uganda [47]. The uncertainty about receiving or the duration of maternity leave could explain this difference as many mothers in the informal sector introduce their babies to other feeds early before they resume work. Despite, these uncertainties, the proportion of employed women in Uganda is increasing with a 9% increase registered between 2011 and 2016 [27]. With the proportion of women in the workforce increasing the inclusion of additional strategies to support breastfeeding is needed for-example, adequate maternity leave, and supportive work environments for day care, both in the formal and informal sector [13].

A higher score in breastfeeding practices at delivery was a positive predictor of EBF. This was likely because women who adhere to recommended feeding practices immediately after birth are more likely to sustain them throughout the breastfeeding period. This is supported by studies done in Haiti, Ethiopia and Ghana where appropriate infant feeding practices such as higher breastfeeding frequency, not giving pre-lacteals and timely initiation of breastfeeding were key determinants of exclusive breastfeeding [35, 48–50].

## Limitations

The use of cluster sampling ensured that the researcher obtained a large enough sample from the visited parishes which allowed for equitable representation of various groups of people. However, it was restricted to only a few parishes limiting generalizability. However, the results can still provide an insight into the role of the informal workplaces on exclusive breastfeeding which could possibly apply to other areas in the country.

This study utilized cross-sectional design which did not allow for temporal attributions to be inferred from the study findings. However, it sets the ground for further investigations using stronger study designs which can infer temporal relationships. Another limitation is that our study assessed EBF based on a mother offering only breast milk in the past 24 hours. This may have overestimated the actual prevalence of EBF as the child might have received other feeds in prior to the recall period as documented by Pullum in 2014 [51]. As data collection was done by visiting households, we may have found only mothers working near their homes who could possibly have a different work environment from those working elsewhere.

## Conclusion

Among mothers working in the informal sector, the factors associated with exclusive breast feeding are number of antenatal care attendance, intention to exclusively breastfeed, proper breastfeeding practices, infant’s age and position at work.

In order to improve EBF among mothers working in the informal sector, the study suggests the following.

### To government

Efforts should be made to protect, promote and support breastfeeding among working mothers in the informal sector as in the formal sector through enforcing the employment act recommendations.

Government should sensitize mothers working in the informal sector about their right to the currently available 60 day leave which provides them with time to establish breastfeeding in the first months of their child’s life.

### To health facilities

Antenatal care services should be focused on building the intention of the mothers to practice exclusive breastfeeding which was found to be a strong predictor of EBF among women in the informal sector.

At ANC, targeted counseling can be offered to mothers working in the informal sector to educate them on how to address the breastfeeding challenges faced while at their work places. Expressing breast milk can be promoted among these mothers to help them attain the 6 months of EBF even when separated from the child for work during the day.

### To employers in the informal sector

Employers in the informal sector should be sensitized through counseling and health education on the benefits of optimal infant feeding and the existing laws governing employee entitlement to maternity leave and breastfeeding support irrespective of their position at the work place.

### To breastfeeding mothers in the informal sector

Mothers should be encouraged to attend antenatal care where they learn about the benefits of exclusive breastfeeding to children below 6 months hence enabling them to make informed decisions about exclusive breastfeeding.

### Further research

Future research is needed to assess the workplaces so as to design and evaluate context specific interventions to support continued exclusive breastfeeding for mothers working in the informal sector.

## Supporting information

S1 Appendix Interviewer administered semi-structured questionnaire. (DOCX)

S2 Appendix Translated interviewer administered semi-structured questionnaire. (DOCX)

S3 Appendix (DOCX)

## Abbreviations

ANC: Antenatal Care
APR: Adjusted Prevalence Ratios
EBF: Exclusive Breastfeeding
Stata: Software for Statistics and Data Science
UDHS: Uganda Demographic Health Survey
UPR: Unadjusted Prevalence Rations
WHO: World Health Organization
