# Supplementary material for: Prevalence of exclusive breastfeeding among mothers in the informal sector, Kampala Uganda
Source: PLoS One. 2020 Sep 24;15(9):e0239062. doi: 10.1371/journal.pone.0239062 (PMC7514031; doi:10.1371/journal.pone.0239062)
Supplement: S1 Appendix — (DOCX) [file pone.0239062.s001.docx]

# **S1Appendix: Interviewer Administered Semi-Structured Questionnaire (English)**

**PREVALENCE OF EXCLUSIVE** **BREASTFEEDING AND ASSOCIATED FACTORS AMONG MOTHERS WORKING THE INFORMAL SECTOR IN KAMPALA DISTRICT-UGANDA**

| **IDENTIFICATION SECTION** | | | | | | | | | |
| --- | --- | --- | --- | --- | --- | --- | --- | --- | --- |
| Questionnaire Number: …………………………… | | | Interview date: ….DD. /…MM. /YYYY……. | | | | | | |
| Interviewer name: …………………….…..……….. | | | Language of interview: ...…………………….….…. | | | | | | |
| Division ……………….…….…………………….. | | | Parish: ……………………………………..……..…. | | | | | | |
| Village/zone: ……………………………….…..…. | | | Ethnicity/Tribe: .………………….…. | | | | | | |
| **Enrolment and eligibility information** | | | | | | | | | |
| 1 | Do you a have any children below 6 months of age in this household? | | | 1. Yes 2. No | | | | | |
| 2 | What is the mother’s occupation? | | | 1. Market vendor 2. Shop attendant (retail/whole sale/hardware /kiosks) 3. Saloonist 4. Restaurants/Bars 5. Agriculture | | | | | |
|  | **SECTION 1: SOCIO-DEMOGRAPHIC, HOUSEHOLD, AND COMMUNITY CHARACTERISTICS** | | | | | | | | |
| 1.1 | What is (index child’s) sex? | | | 1. Male 2. Female | | | | | |
| 1.2 | What is (index child’s) age in months? | | | ………………months | | | | | |
| 1.3 | What is (index child’s) date of births? | | | DD/MM/YYYY.(Ask for birth certificate) | | | | | |
| 1.4 | How old are you (in completed years)? | | | ……..….years (Ask for national ID) | | | | | |
| 1.5 | Who is the head of this household? What is the sex of the household head? | | | 1. Male 2. Female | | | | | |
| 1.6 | How are you related to the household head? | | | 1. Spouse 2. Parent 3. Boss 4. Other (specify)……………………. | | | | | |
| 1.7 | What is your Marital status? | | | 1. Single/living alone…..…skip to 1.10 2. Divorced/separated….....skip to 1.10 3. Widowed…………...…..skip to 1.10 4. Married/cohabiting | | | | | |
| 1.8 | What is the highest level Education your spouse? | | | 1. No formal schooling 2. Primary (P.1-P.7) 3. Secondary 4. Tertiary | | | | | |
| 1.9 | What is your spouse’s employment? | | | …………………………………… | | | | | |
| 1.10 | What is your highest level of Education attained? | | | 1. No formal schooling 2. Primary (P.1-P.7) 3. Secondary 4. Tertiary | | | | | |
| 1.11 | What is your Religion? | | | 1. Catholic 2. Protestant 3. Muslim 4. Pentecost (Born again) 5. Others (specify)………………… | | | | | |
| 1.12 | How many children do you have? | | | …………………………….…………… | | | | | |
| 1.13 | How old is the child before (index child)? | | | …………………………months | | | | | |
|  | **SECTION 2: OWNERSHIP OF HOUSEHOLD ASSETS & INCOME** | | | | | | | | |
| 2.1 | Do you/anyone in your house hold possess the following?  Tick where appropriate. | | | Yes | | | No | | |
| A | Electricity | | |  | | |  | | |
| B | Working radio | | |  | | |  | | |
| C | Working TV | | |  | | |  | | |
| D | Working computer | | |  | | |  | | |
| E | Working mobile telephone | | |  | | |  | | |
| F | Fixed phone | | |  | | |  | | |
| G | Working fridge | | |  | | |  | | |
| H | Working bicycle | | |  | | |  | | |
| I | Working motorcycle/scooter | | |  | | |  | | |
| J | Working car/truck | | |  | | |  | | |
| K | Working generator | | |  | | |  | | |
| L | Working solar panel | | |  | | |  | | |
| M | Working gas stove | | |  | | |  | | |
| N | At least 2 sets of shoes, clothes | | |  | | |  | | |
| O | Agricultural land(anywhere) | | |  | | |  | | |
| 2.2 | **HOUSING MATERIAL (OBSERVE)** | | | | | | | | |
| A | Floor material | | | 1. Mud 2. Concrete/Bricks /Tiles/stone/wood 3. Other (specify)…………………… | | | | | |
| B | Wall material | | | 1. Straw and thatched 2. Mud& poles, unburnt brinks 3. cement, burnt bricks, timber, stone) 4. Other (specify)…………………… | | | | | |
| C | Roof material | | | 1. Straw and thatched 2. Iron sheets 3. Wood/Concrete/tiles/asbestos 4. Other (specify)…………….…… | | | | | |
|  | **SECTION 3: EMPLOYMENT FACTORS** | | | | | | | | |
| 3.1 | What is your position at work? | | | 1. Owner 2. Manager/ 3. Other (specify): ………………….. | | | | | |
| 3.2 | How far is your work place from home? | | | ………Kilometers | | | | | |
| 3.3 | Did you get/are you on maternity leave? | | | 1. Yes 2. No…………...…......skip to 3.9 | | | | | |
| 3.4 | Is/Was the maternity leave paid? | | | 1. Yes 2. No | | | | | |
| 3.5 | How long was /is it? | | | ………….days **/** ………months | | | | | |
| 3.6 | How long after delivery did you return to work? | | | ………..days **/** ……...…months | | | | | |
| 3.7 | In your view is/was this period sufficient? | | | 1. Yes 2. No | | | | | |
| 3.8 | What time do you normally go to work? | | | ………………………………………… | | | | | |
| 3.9 | What time do you normally leave work? | | | ………………………………………… | | | | | |
| 3.10 | Is this how you spend your day yesterday? | | | 1. Yes 2. No | | | | | |
| 3.11 | What made it different? | | | ………………………………………. | | | | | |
| 3.12 | Did your spouse get leave after you delivered? | | | ……………………………………….. | | | | | |
| 3.13 | How long did your spouse take to return to work after you delivered | | | ………..days **/** ………months | | | | | |
| 3.14 | Have you resumed work? | | | 1. Yes 2. No……………….skip to 3.18 | | | | | |
| 3.15 | Do you take your child to work? | | | 1. Yes 2. No………………….skip to 3.18 | | | | | |
| 3.16 | How do you maintain your child at work? (describe where she puts the child and how she feeds him/her) | | | …………………………………………………………………………………………………………………………….. | | | | | |
| 3.17 | If you are not there to feed the baby, what type of food is the baby fed? | | | 1. Expressed breast milks 2. Other milks 3. Other (specify)……………… | | | | | |
| 3.18 | If you are not there to feed the baby, How is (index child) fed? | | | 1. Bottle 2. Cup &/ Spoon 3. Other (specify)……………………. | | | | | |
| 3.19 | When you are not home or cannot feed (index child) yourself, who does it? | | | …………………………………………………………………………………… | | | | | |
| 3.20 | Do you have the following at work? (tick off where applicable) | | | 1. Private place to breastfeed/pump 2. Place to store breast milk 3. Place to keep the baby as you work 4. None | | | | | |
|  | **SECTION 4: DELIVERY CHARACTERISTICS AND HEALTH RELATED FACTORS** | | | | | | | | |
| 4.1 | Did you attend ANC? | | | 1. Yes 2. No………………..skip to 4.3 | | | | | |
| 4.2 | If Yes, How many times? | | | 1. 0 2. 1 3. 2-3 4. ≥4 | | | | | |
| 4.3 | Where did you of deliver this child from? | | | 1. Public 2. Home 3. Private 4. Other (specify)………………… | | | | | |
| 4.4 | What was the mode of delivery | | | 1. Normal 2. Cesarean | | | | | |
| 4.5 | Was the baby left in your room after delivery? | | | 1. Yes 2. No | | | | | |
| 4.6 | What was the number of children born, when index child was born? | | | 1. Singleton 2. Twins /multiple | | | | | |
| 4.7 | Did (index child) have any birth complications (Tick all that apply) | | | 1. Premature 2. Crying/breathing problem at birth 3. Sepsis 4. Low birth weight 5. Other (specify)………………... | | | | | |
| 4.8 | Do you have any birth/ health complications | | | 1. Yes 2. No | | | | | |
| 4.9 | Which ones? ***List all mother mentions*** | | | …………………………………………… | | | | | |
| 4.10 | Did you ever receive Counselling and Education on Exclusive Breast feeding? | | | 1. Yes 2. No………………...skip to 4.12 | | | | | |
| 4.11 | What was discussed? | | | 1. Initiation 2. Positioning 3. Frequency 4. Others (specify) ………………… | | | | | |
| 4.12 | What is your single major source of breastfeeding information? | | | 1. Health worker 2. Family/Peers 3. Media 4. Other (specify) ………………… | | | | | |
|  | **SECTION 5: INFANT FEEDING PRACTICES & HEALTH STATUS** | | | | | | | | |
| 5.1 | In the first three days after delivery, was he/she given anything to drink other than breast milk? | | | 1. Yes 2. No ….…………..….. skip to 5.3 | | | | | |
| 5.2 | What was s/he given to drink? Anything else? (*list what mother says*) | | | …………………………………………………………………………………… | | | | | |
| 5.3 | How long after birth did you first put (index child) to the breast? | | | 1. Immediately (less than 1 hour) 2. ……….. hours 3. …………days | | | | | |
| 5.4 | Who was responsible for doing this? | | | 1. Self 2. Health worker 3. Care takers 4. Other (specify)………………..….. | | | | | |
| 5.5 | Are you still breastfeeding to (index child)? | | | 1. Yes …………..……..skip to 5.8 2. No | | | | | |
| 5.6 | For how many months did you breastfeed index child? | | | …………………….months | | | | | |
| 5.7 | Why did you stop breastfeeding index child? | | | …………………………………………………………………………………… | | | | | |
| 5.8 | How many times did you breastfeed [index child] yesterday? | | | 1. None 2. 1 -3 3. 4-7 4. 8+ | | | | | |
| 5.9 | Yesterday, during the day or night did (index child) received another feeds besides breast milk? | | | 1. Yes 2. No………………....skip to 5.15 | | | | | |
| 5.10 | Yesterday, during the day or night, did (index child) consume any of the following foods. | | | 1. Vitamins /medicines, mineral supplements 2. Grains, roots, tubers 3. Legumes, nuts 4. Diary(milk, yoghurt, cheese) 5. Flesh foods (meat, fish, poultry, offal) 6. Eggs 7. Vitamin A rich fruits & vegetables 8. Other (specify) …………………. | | | | | |
| 5.11 | Aside from breastfeeding, How many times did index child receive other feeds yesterday? | | | …………………..……………………..………………………………………… | | | | | |
| 5.12 | When did you start giving other feeds? (months) | | | ………………………………………… | | | | | |
| 5.13 | Since this time yesterday, has [index child] been given anything to drink from a bottle with a nipple or teat? | | | 1. Yes 2. No………………….skip to 5.16 3. Don’t know | | | | | |
| 5.14 | What was given from the bottle? | | | ……………………………………...…………………………….…………………. | | | | | |
| 5.15 | Who decided that (index child) be fed this way? | | | 1. Health worker 2. Self 3. Husband/Grand parent 4. Others (specify)……...……… | | | | | |
| 5.16 | What was your/their main reason for this decision? | | | ………………………………………………………………………………………… | | | | | |
| 5.17 | Has [index child] been sick in the last 2 weeks? | | | 1. Yes 2. No ……………….skip to 6.1 | | | | | |
| 5.18 | What has she been sick of in the last 2 weeks?  **NOTE:** (***diarrhea is if the child has had more than 3 loose stools in the last 24hours)*** | | | 1. Diarrhea 2. Fever 3. Cough 4. Other (specify) …………….…… | | | | | |
| 5.19 | During [child index’s] sickness, did [index child] breastfeed less than usual, the same, or more? | | | 1. Less than usual 2. About the same 3. More than usual 4. Don’t Know | | | | | |
| 5.20 | Has [index child] recovered from the sickness in the past 2 weeks? | | | 1. Yes 2. No | | | | | |
| 5.21 | In the days after [index child]’s sickness, has s/he breastfed/ate less than usual, the same, or more? | | | 1. Less than usual 2. About the same 3. More than usual 4. Don’t Know | | | | | |
|  | **SECTION 6: MATERNAL KNOWLEDGE, ATTITUDES AND BELIEFS ON EBF** | | | | | | | | |
|  | **KNOWLEDGE** | | | | | | | | |
| 6.1 | What is the first food a newborn baby should receive? | | | 1. Only breast milk 2. Other ………………….. 3. Don’t know | | | | | |
| 6.2 | Breast milk alone without even water can sustain the baby for six months? | | | 1. Yes 2. No | | | | | |
| 6.3 | What are the benefits for a baby if he or she receives only breast milk during the first six months of life? ***(list any 3)*** | | | …………………………………………………………………………………………………….……………………………….. | | | | | |
| 6.4 | What are the physical or health benefits for a mother if she exclusively breastfeeds her baby?(Probe if necessary) **(list any 3)** | | | ……………………………………………………………………………………………………………………………………... | | | | | |
| 6.5 | How long should a baby receive nothing more than breast milk? ***(Probe if necessary)*** | | | ………………………………………………………………………………………… | | | | | |
| 6.6 | How often should a baby younger than six months be breastfed / fed with breast milk? | | | 1. On demand 2. Other 3. Don’t know | | | | | |
|  | **BREASTFEEDING BEHAVIOURAL BELIEFS** | | |  | | | | | |
| 6.7 | Do you think you could give only breast milk (without water, nor any other liquid/feeds to the baby for 6 months? | | | 1. Yes 2. No .………………….skip to 6.10 | | | | | |
| 6.8 | Why do you think you can/can ́t give (or would not be able to give) only breast milk to the baby for 6 months? | | | ………………………………………………………………………………………… | | | | | |
|  | **Attitude** | | | | | | | | |
| 6.9 | It is good to exclusively breastfeed your baby for six months | | | 1. Not good 2. Not sure 3. Good………………...skip to 6.12 | | | | | |
| 6.10 | Can you tell me the reasons why you think it is not good? | | | ………………………………………………………………………………………… | | | | | |
| 6.11 | It is not difficult to exclusively breastfeed my baby for six months | | | 1. Not difficult 2. So-so 3. Difficult …..skip to 6.14 | | | | | |
| 6.12 | Can you tell me the reasons why you think it is difficult? | | | ………………………………………………………………………………………… | | | | | |
| 6.13 | I feel confident when breast-feeding my child | | | 1. Not confident ...……..skip to 6.16 2. Ok/ so-so 3. Confident | | | | | |
| 6.14 | Can you tell me the reasons why you do not feel confident? | | | ………………………………………………………………………………………… | | | | | |
| 6.15 | I can confidently express breastmilk for my child | | | 1. Not confident 2. Ok/ so-so 3. Confident | | | | | |
| 6.16 | Can you tell me the reasons why you feel /do not feel confident? | | | ………………………………………………………………………………………… | | | | | |
|  | **INTENTION** | | | | | | | | |
| 6.17 | Think back before you had your baby, how long did you intend to exclusively breastfeed him/her? | | | …………………………………………………………………………..…………… | | | | | |
|  | **CHALLENGES IN PRACTICING EXCLUSSIVE BRESTFEEDING** | | | | | | | | |
| 6.18 | What challenges do/did you face when giving only breast milk to index child? | | | …………………………………………………………………… | | | | | |
| 6.19 | How do/did you deal with these challenges? | | | …………………………………………………………………… | | | | | |
| Have the following been a challenge to you, rate how much of a challenge they have been to you **(**1=*not at all*, 2=*small problem* ,3= *neutral*, 4=*big problem,* 5=*very big problem (read out question and circle accordingly )* | | | | | | | | | |
| 6.20 | | Lack of a private place to breastfeed | | | **1** | **2** | **3** | **4** | 5 |
| 6.21 | | Less time to feed for the child | | | **1** | **2** | **3** | **4** | 5 |
| 6.22 | | Staying far away from the child too long | | | **1** | **2** | **3** | **4** | 5 |
| 6.23 | | Lack of support from significant others (husband, parents, employers) | | | **1** | **2** | **3** | **4** | 5 |
| 6.24 | | Lack of professional support | | | **1** | **2** | **3** | **4** | 5 |

***Thank you for your participation***
