# Supplementary material for: Prevalence of exclusive breastfeeding among mothers in the informal sector, Kampala Uganda
Source: PLoS One. 2020 Sep 24;15(9):e0239062. doi: 10.1371/journal.pone.0239062 (PMC7514031; doi:10.1371/journal.pone.0239062)
Supplement: S2 Appendix — (DOCX) [file pone.0239062.s002.docx]

# **S2 Appendix: Interviewer Administered Semi-Structured Questionnaire (Luganda)**

| **IDENTIFICATION SECTION** | | | | | | | | |
| --- | --- | --- | --- | --- | --- | --- | --- | --- |
| Questionnaire Number: …………………… | | Interview date: ….DD. /…MM. /YYYY……. | | | | | | |
| Interviewer name: …………………….…….. | | Language of interview: ……..……………. | | | | | | |
| Division ……………….…….……………….. | | Parish: ……………………….…..……..…. | | | | | | |
| Village/zone: …………………………………. | | Tribe /ethnicity: ....…………………....…. | | | | | | |
| **Enrolment and eligibility information** | | | | | | | | |
| 1 | Do you a have any children below 6 months of age?  **Olina owana ali wansi we’myezi 6?** | | 1. Yes 2. No | | | | | |
| 2 | What is the mother’s occupation?  **Okola mulimuki?** | | 1. Market vendor 2. Shop attendant (retail/whole sale/hardware /kiosks) 3. Saloonist 4. Restaurants 5. Agriculture | | | | | |
|  | **SECTION 1: SOCO-DEMOGRAPHIC, HOUSEHOLD, AND COMMUNITY CHARACTERISTICS** | | | | | | | |
| 1.1 | What is the sex of the index child?  **Owana ono wakikula ki?** | | 1. Male 2. Female | | | | | |
| 1.2 | What is (index child’s) date of births?  **Owana ono yazalibwa di?** | | DD/MM/YYYY.(*Ask for birth certificate*) | | | | | |
| 1.3 | What is (index child’s) age in months?  **Owana ono alina emyezi emeka?** | | ………………months | | | | | |
| 1.4 | How old are you (in completed years)?  **Oweza emyaka emmeka emijjuvu?** | | ……….years (Ask for national ID) | | | | | |
| 1.5 | Who is the head of this household? What is the sex of the household head?  **Omukulu wamaka ganno yanni ki?** | | 1. Male 2. Female | | | | | |
| 1.6 | How are you related to the household head?  **Oyita otya omukulu wamaka ganno?** | | 1. Spouse 2. Parent 3. Other (specify) ..…………. | | | | | |
| 1.7 | What is your Marital status?  **Oli muffumbo?** | | 1. Single/living alone.…skip to 1.9 2. Divorced/separated…skip to 1.9 3. Widowed…………...skip to 1.9 4. Married/cohabiting | | | | | |
| 1.8 | What is the highest level Education your spouse?  **Omwami yasoma ko paka kubina ki?** | | 1. No formal schooling 2. Primary (P.1-P.7) 3. Secondary 4. Tertiary | | | | | |
| 1.9 | What is your spouse’s employment?  **Omwami akola mulimu ki?** | | …………………………………………………………………………. | | | | | |
| 1.10 | What is your highest level of Education attained?  **Wasoma ko paka kubina ki?** | | 1. No formal schooling 2. Primary (P.1-P.7) 3. Secondary 4. Tertiary | | | | | |
| 1.11 | What is your Religion?  **Oliwa ddini ki?** | | 1. Catholic 2. Protestant 3. Muslim 4. Pentecost (Born again) 5. Others (specify)…………… | | | | | |
| 1.12 | How many children do you have?  **Olina abanna bamekka?** | | …………………………….…… | | | | | |
| 1.13 | How old is the child before (index child)?  **(Index child) gwadako yekana ki obukulu?** | | …………………………months | | | | | |
|  | **SECTION 2: OWNERSHIP OF HOUSEHOLD ASSETS & INCOME** | | | | | | | |
| 2.1 | Do you/anyone in your house hold possess the following? **Olina/omutu yenna mumaka ganno alina ebitu bino?** | | Yes | | | No | | |
| A | Electricity | |  | | |  | | |
| B | Working radio | |  | | |  | | |
| C | Working TV | |  | | |  | | |
| D | Working computer | |  | | |  | | |
| E | Working mobile telephone | |  | | |  | | |
| F | Fixed phone | |  | | |  | | |
| G | Working fridge | |  | | |  | | |
| H | Working bicycle | |  | | |  | | |
| I | Working motorcycle/scooter | |  | | |  | | |
| J | Working car/truck | |  | | |  | | |
| K | Working generator | |  | | |  | | |
| L | Working solar panel | |  | | |  | | |
| M | Working gas stove | |  | | |  | | |
| N | At least 2 sets of shoes, clothes | |  | | |  | | |
| V | Agricultural land | |  | | |  | | |
| 2.2 | **HOUSING MATERIAL (OBSERVE)** | | | | | | | |
| A | Floor material | | 1. Mud 2. Concrete/Bricks/Tiles/stone/wood 3. Other (specify)………………… | | | | | |
| B | Wall material | | 1. Cement, burnt bricks, timber, stone) 2. Mud& poles, unburnt brinks 3. Straw and thatched 4. Other (specify)………………… | | | | | |
| C | Roof material | | 1. Wood/Concrete/tiles/asbestos 2. Iron sheets 3. Straw and thatched 4. Other (specify)………………… | | | | | |
|  | **SECTION 3: EMPLOYMENT FACTORS** | | | | | | | |
| 3.1 | What is your position at work?  **Kumulimu gwo, olina bufunanizibwa ki?** | | 1. Owner 2. Manager 3. Other (specify):..………….. | | | | | |
| 3.2 | How far is your work place from home?  **Buwanfu ki obuli Okuva wokolera okutuka wobeera?** | | ………Kilometers | | | | | |
| 3.3 | Did you get/are you on maternity leave?  **Wafunna “maternity leave”?** | | 1. Yes 2. No………......skip to 3.9 | | | | | |
| 3.4 | Is/Was the maternity leave paid?  **Wali/Ofunna/ omusala mu “maternity leave”?** | | 1. Yes 2. No | | | | | |
| 3.5 | How long was /is it?  **Egenda kutyala/Watyala banga ki?** | | ………….days **/** ………months | | | | | |
| 3.6 | How long after delivery did you return to work?  **Watwala banga ki okudayo kumulimu nga omazze okuzala?** | | ………..days **/** ……...…months | | | | | |
| 3.7 | In your view is/was this period sufficient?  **Mukulaba kwo, akesera kanno kali kamala?** | | 1. Yes 2. No | | | | | |
| 3.8 | What time do you normally go to work?  **Otera, genda sawa meka kumulimu?** | | ………………………………………………………………………… | | | | | |
| 3.9 | What time do you normally leave work?  **Otera, kuva sawa meka kumulimu?** | |  | | | | | |
| 3.10 | Is this how you spend your day yesterday? **Byotyo bwewakoze jjo?** | | 1. Yes 2. No | | | | | |
| 3.11 | What made it different?  **Ki ekyabadde ekyenjawulo?** | | ………………………………………………………………………… | | | | | |
| 3.12 | Did your spouse get leave after you delivered?  **Omwami yafunna a kadde nga takola oluvanyuma lwo kuzala?** | | 1. Yes 2. No | | | | | |
| 3.13 | How long did your spouse take to return to work after you delivered?  **Omwami wo kyamutwala banga ki okudayo kumulimu nga omazze okuzalla?** | | ………..days **/** ………months | | | | | |
| 3.14 | Have you resumed work?  **Watandinsse okola?** | | 1. Yes 2. No | | | | | |
| 3.15 | Do you take your child to work?  **(Index child) ogenda naye kumulimu?** | | 1. Yes 2. No…………….skip to 3.14 | | | | | |
| 3.16 | How do you maintain your child at work? (describe where she puts the child and how she feeds him/her)  **(Index child) omulabilira otya nga olikumulimu?** | | ………………………………………………………………………………………………………………………………………………….. | | | | | |
| 3.17 | If you are not there to feed the baby, what type of food is the baby fed?  **Bwoba toliwo okuyonsa (index child), kyakulya ki kyafuna?** | | 1. Expressed breast milks 2. Other milks 3. Other (specify)…………… | | | | | |
| 3.18 | If you are not there to feed the baby, How is index child fed?  **Bwoba toliwo okuyonsa (index child), bamulisisa muki?** | | 1. Bottle 2. Cup &/ Spoon 3. Other (specify)…………. | | | | | |
| 3.19 | When you are not home or cannot feed the baby yourself, who does it?  **Bwoba toliwo okuyonsa (index child), ani amulisa?** | | ………………………………………………………………………… | | | | | |
| 3.20 | Do you have the following at work? (tick off where applicable)  **Wokolera mulina kubintu binno?** | | 1. Private place to breastfeed/pump 2. Place to store breast milk 3. Place to keep the baby 4. None | | | | | |
|  | **SECTION 4: DELIVERY CHARACTERISTICS AND HEALTH RELATED FACTORS** | | | | | | | |
| 4.1 | Did you attend ANC?  **Wagenda kudwaliro nga olilubuto?** | | 1. Yes 2. No……………..skip to 4.3 | | | | | |
| 4.2 | If Yes, How many times?  **Wagenda yo emirundi emeka?** | | 1. 0 2. 1 3. 2-3 4. ≥4 | | | | | |
| 4.3 | Where did you of deliver this child from?  **Wazalira wa?** | | 1. Public 2. Home 3. Private 4. Other (specify)…………… | | | | | |
| 4.4 | What was the mode of delivery  **Wazala oytya?** | | 1. Normal 2. Cesarean | | | | | |
| 4.5 | Was the baby left in your room after delivery?  **(Index child) yasigala mukasenge nawe naga ozadde?** | | 1. Yes 2. No | | | | | |
| 4.6 | What was the number of children born, when index child was born?  **Kulyo, wazala bana bameka?** | | 1. Singleton 2. Twins /multiple | | | | | |
| 4.7 | Did (index child) have any birth complications (Tick all that apply)  **(Index child) yalina ko obusibu bwona nga wakamuzala?** | | 1. Premature 2. Crying/breathing problem at birth 3. Sepsis 4. Low birth weight 5. Other (specify)……………... | | | | | |
| 4.8 | Do you have any birth/ health complications?  **Olina bukosefu byona byewafuna nga ozadde?** | | 1. Yes 2. No | | | | | |
| 4.9 | Which ones? ***List all mother mentions***  **Buliwa?** | | ……………………………………………………………………………………………… | | | | | |
| 4.10 | Did you ever receive Counselling and Education on Exclusive Breast feeding?  **Waffuna ko mumisomo ku nyonza yabanna nga tobawa kintukila kyona?** | | 1. Yes 2. No…………...skip to 5.1 | | | | | |
| 4.11 | What was discussed?  **Bakusomesa yo ki?** | | 1. Initiation 2. Positioning 3. Frequency 4. Others (specify) ……..……… | | | | | |
| 4.12 | What is your single major source of information?  **Okusoma kunno wakufuna wa?** | | 1. Health worker 2. Family/Peers 3. Media 4. Other (specify)…………….… | | | | | |
|  | **SECTION 5: INFANT FEEDING PRACTICES & HEALTH STATUS** | | | | | | | |
| 5.1 | In the first three days after delivery, was he/she given anything to drink other than breast milk?  **Munaku esatu esasoka, index child yafunnako ebyokunya/ebyokulya ebirala byonna okujako ebere?** | | 1. Yes 2. No ………..….. skip to 5.3 | | | | | |
| 5.2 | What was s/he given to drink? Anything else? **(*list what mother says*)**  **Ki kyeyafuna?** | | ……………………………………………………………………………………………………………… | | | | | |
| 5.3 | How long after birth did you first put (index child) to the breast?  **Wayitawo kabanga ki okutekka (index child) kubere?** | | 1. Immediately (less than 1 hour) 2. ……….. hours 3. …………days | | | | | |
| 5.4 | Who was responsible for doing this?  **Ani yamutekako?** | | 1. Self 2. Health worker 3. Care takers 4. Other (specify)…………..….. | | | | | |
|  | Did you ever try to breastfeed?  **Wali oyosenzako?** | | 1. Yes 2. No | | | | | |
| 5.5 | Are you still breastfeeding to (index child)?  **Index child akyayonka?** | | 1. Yes.…………..skip to 5.8 2. No | | | | | |
| 5.6 | For how many months did you breastfeed index child?  **Wamuyonsa kumala kisera ki?** | | …………………….months | | | | | |
| 5.7 | Why did you stop breastfeeding index child?  **Lwaki walekera awo?** | | ………………………………………………………………………… | | | | | |
| 5.8 | How many times did you breastfeed [index child] yesterday?  **Egulo, (index child) yayonse emirubdi emeka?** | | 1. None 2. 1 -3 3. 4-7 4. 8+ | | | | | |
| 5.9 | Yesterday, during the day or night did (index child) received another feeds besides breast milk?  **Egulo, yafunye yo ekykunya/ekyokulya ekilala okujako eberre?** | | 1. Yes 2. No……….……....skip to 5.15 | | | | | |
| 5.10 | Yesterday, during the day or night, did (index child) consume any of the following foods.  **Egulo, [Index child]** **yafunye yo kubyokulya bino?** | | 1. Vitamins /medicines, mineral supplements 2. Grains, roots, tubers 3. Legumes, nuts 4. Diary(milk, yoghurt, cheese) 5. Flesh foods (meat, fish, poultry, offal) 6. Eggs 7. Vitamin A rich fruits & vegetables 8. Other (specify) ………….…. | | | | | |
| 5.11 | Aside from breastfeeding, How many times did index child receive other feeds yesterday?  **Nga ogyeko okuyonsa, index child yanwenda/yalidde emirudi emeka?** | | …………………..……………………..…………………………………………….………………… | | | | | |
| 5.12 | When did you start giving other feeds?  **Watandika di okumuwa ebyokunya/ebyokula?** | | MMM……/….YYYY | | | | | |
| 5.13 | Since this time yesterday, during the day or night has [index child] been given anything to drink from a bottle with a nipple or teat?  **Egulo, [Index child]** **yanweledeko ku nyanto?** | | 1. Yes 2. No…………….skip to 5.15 3. Don’t know | | | | | |
| 5.14 | What was given from the bottle?  **Ki kyeyanyelede mu?** | | ……………………………………...……………………………….… | | | | | |
| 5.15 | Who decided that (index child) be fed this way?  **Ani yasalawo kundiisa ya index child?** | | 1. Health worker 2. Self 3. Husband/Grand parent 4. Others (specify)……...……… | | | | | |
| 5.16 | What was your/their main reason for this decision?  Lwaki wa/yaslawo bwati? | | ………………………………………………………………………… | | | | | |
| 5.17 | Has [index child] been sick in the last 2 weeks?  **[Index child]** **yalwalako musabitti birri eziiyisse?** | | 1. Yes 2. No ………..……....skip to 5.24 | | | | | |
| 5.18 | What has she been sick of in the last 2 weeks?  **Ki ekibadde kimuluma?**  **NOTE:** (***diarrhea is if the child has had more than 3 loose stools in the last 24hours)*** | | 1. Diarrhea 2. Fever 3. Cough 4. Other (specify) ……….…… | | | | | |
| 5.19 | During [child index’s] sickness, did [index child] breastfeed less than usual, the same, or more?  **Mukisera waberedde nga mulwadde, akyusiza mu mukunyonka?** | | 1. Less than usual 2. About the same 3. More than usual 4. Don’t Know | | | | | |
| 5.20 | Has [index child] recovered from the sickness in the past 2 weeks?  **Yawona?** | | 1. Yes 2. No ………...…….skip to 5.24 | | | | | |
| 5.21 | In the days after [index child]’s sickness, has s/he breastfed/ate less than usual, the same, or more?  **Mukisera nga yakawona wamunyonsezza otya?** | | 1. Less than usual 2. About the same 3. More than usual 4. Don’t Know | | | | | |
|  | **SECTION 6: MATERNAL KNOWLEDGE, ATTITUDES AND BELIEFS ON EBF** | | | | | | | |
|  | **KNOWLEDGE** | | | | | | | |
| 6.1 | What is the first food a newborn baby should receive?  **Omwana eyakazalibwa alina kufuna kyakunwa/lya ki?** | | 1. Only breast milk 2. Other …………….. 3. Don’t know | | | | | |
| 6.2 | Breast milk alone without even water can sustain the baby for six months?  **Amabere goka gayinza okubezzawo omwana okumala emyezi 6?** | | 1. Yes 2. No | | | | | |
| 6.3 | What are the benefits for a baby if he/she receives only breast milk during the 1^st^ 6 months of life? ***(list any 3)***  **Migaso ki egili mukunyonsa kyoka okumala emwezi 6 (wandiikayo satu)?** | | ……………………………………………………………………………………………………………… | | | | | |
| 6.4 | What are the physical or health benefits for a mother if she exclusively breastfeeds her baby?(Probe if necessary) (list any 3)  **Migaso ki mama gyafunna mukunyonsa omwana okumala emwezi 6?** | | ……………………………………………………………………………………………………………………………………………………. | | | | | |
| 6.5 | How long should a baby receive nothing more than breast milk? ***(Probe if necessary)***  **Owana yandiimaze bangaki naga affuna mabere goka?** | | ………………………………………………………………………… | | | | | |
| 6.6 | How often should a baby younger than six months be breastfed or fed with breastmilk? | | 1. On demand 2. Other 3. Don’t know | | | | | |
|  | **BREASTFEEDING BEHAVIOURAL BELIEFS** | |  | | | | | |
| 6.7 | Do you think you could give only breast milk (without water, nor any other liquid/feeds to the baby for 6 months?  **Olowoza osobola okuyonsa kyoka nga omwana tomuwa kyakunya.kyakulya kilalakona?** | | 1. Yes 2. No ….……..skip to 6.10 | | | | | |
| 6.8 | Why do you think you can ́t give (or would not be able to give) only breast milk to the baby for 6 months?  **Lwaki olowaosa kisibu?** | | ……………………………………………………………………………………………………………… | | | | | |
|  | **Attitude** | | | | | | | |
| 6.9 | How good do you think it is to breastfeed your baby exclusively for six months?  **Olowoza kilungi okuyonsa obuyonsa okumala emyezi 6?** | | 1. Not good 2. Not sure 3. Good……..skip to 6.12 | | | | | |
| 6.10 | Can you tell me the reasons why it is not good?  **Lwaki olowosa sikilungi?** | | ……………………………………………………………………………… | | | | | |
| 6.11 | How difficult is it for you to breastfeed your baby exclusively for six months?  **Kikusibuwalila okuyonsa owana nga tolina kyomuwa kyona?** | | 1. Not difficult....skip to 6.14 2. So-so 3. Difficult | | | | | |
| 6.12 | Can you tell me the reasons why it is difficult?  **Lwaki kikusibuwalila?** | | ……………………………………………………………………………… | | | | | |
|  | **SELF-EFFICACY & CONTROL** | | | | | | | |
| 6.13 | How confident do you feel in breastfeeding your child?  **Wewulira otya ku bukugu, byolina mukunyonsa?** | | 1. Not confident 2. Ok/ so-so 3. Confident …skip to 6.16 | | | | | |
| 6.14 | Can you tell me the reasons why you do not feel confident?  **Lwaki tewewulia nti osobola?** | | ……………………………………………………………………………… | | | | | |
| 6.15 | How confident do you feel in expressing and storing breast milk so that someone else can feed your baby?  **Wewulira otya ku bukugu, byolina okukamula amata, omutu omulala asobole okkugawa amwana wo?** | | 1. Not confident 2. Ok/ so-so 3. Confident ...skip to 6.18 | | | | | |
| 6.16 | Can you tell me the reasons why you do not feel confident?  **Lwaki tewewulia nti osobola?** | | ……………………………………………………………………………………………………………… | | | | | |
|  | **INTENTION** | | | | | | | |
| 6.17 | Think back before you had your baby, did you intend to exclusively breastfeed him/her for 6 months?  **Bwewali olubuto, wali wasalawo okuyonsa nga tomuwa kintu kilala kyona okuma emyezi 6** | | ……………………………………………………………………………………………………………… | | | | | |
|  | **CHALLENGES IN PRACTICING EXCLUSSIVE BRESTFEEDING** | | | | | | | |
| 6.18 | What challenges do/did you face when giving only breast milk to index child?  **Obuzibu ki byofunye mukunyonza?** | | ………………………………………………………………………………………………….…………… | | | | | |
| 6.19 | How do/did you deal with these challenges?  **Obuvunusse otya?** | | ………………………………………………………………………… | | | | | |
| Have the following been a challenge to you, rate how much of a challenge they have been to you **(**1=*not at all*, 2=*mild* ,3= *moderate*, 4=*severe,* 5=*unbearable(read out question and circle accordingly )*  ***Waliwo ebimu kumanga ebikalubiriza mukuyonsa? Bikalubiriza buzito ki?*** | | | | | | | | |
| 6.20 | Lack of a private place to breastfeed/express  **Obutaba nakifo kyekusifu ewokunyoseza/okamula amata** | | | **1** | **2** | **3** | **4** | 5 |
| 6.21 | Less time to feed for the child  **Obutaba nabudde bumala kuyonsa o’mwana** | | | **1** | **2** | **3** | **4** | 5 |
| 6.22 | Staying far away from the child too long  **Obutaba nabudde bumala no’mwana** | | | **1** | **2** | **3** | **4** | 5 |
| 6.23 | Lack of support from significant others (husband, parents, employers)  **Obutaba na buyambi akuva mubanange** | | | **1** | **2** | **3** | **4** | 5 |
| 6.24 | Lack of professional support  **Obutaba nabunyanbi akuva mubakugu** | | | **1** | **2** | **3** | **4** | 5 |

**The END. *Thank you for your time***
