# Supplementary material for: Prevalence of exclusive breastfeeding among mothers in the informal sector, Kampala Uganda
Source: PLoS One. 2020 Sep 24;15(9):e0239062. doi: 10.1371/journal.pone.0239062 (PMC7514031; doi:10.1371/journal.pone.0239062)
Supplement: S3 Appendix — (DOCX) [file pone.0239062.s003.docx]

**Prevalence of Exclusive Breastfeeding among Mothers in the Informal Sector, Kampala Uganda**

Key variables studied

| Number | Variable name |
| --- | --- |
| 1 | Place of residence |
| 2 | Occupation of mother |
| 3 | Position at work |
| 4 | Age of child in months |
| 5 | Sex of child |
| 6 | Birth order |
| 7 | Tribe of mother in years |
| 8 | Age of mother |
| 9 | Education level of mother |
| 10 | House hold Head |
| 11 | Sex of Household head |
| 12 | Relationship to house hold head |
| 13 | Marital status |
| 14 | Employment of spouse |
| 15 | Education level of spouse |
| 16 | Number of children |
| 17 | Previous birth interval |
| 18 | Distance of work from home |
| 19 | Received Maternity leave |
| 20 | Duration of maternity leave |
| 21 | Age of infant when mother resumed work |
| 22 | Average time spent at work daily |
| 23 | Paternity Leave for spouse |
| 24 | Duration of paternity leave |
| 25 | Spouse assists feeding child while on leave |
| 26 | Takes child to work |
| 27 | Breastfeeding facilities at work |
| 28 | Feeding option at work |
| 29 | Still breastfeeding? |
| 30 | If no, Why |
| 31 | If no, Who feeds child when separated |
| 32 | If no, Feeding method |
| 33 | Age of initiation of bottle feeding /other feeds |
| 34 | Duration of EBF if no |
| 35 | Expresses breast milk |
| 36 | Ever Expressed |
| 37 | If yes, Reason for Expressing |
| 38 | Frequency of Breastfeeding |
| 39 | Counseling and education on breastfeeding |
| 40 | Source of breastfeeding information |
| 41 | Intention to EBF |
| 42 | Delivery mode |
| 43 | Time to Initiation of EBF |
| 44 | Supported to breastfeed at birth by health worker |
| 45 | Rooming in |
| 46 | Skin to skin contact |
| 47 | Frequency of feeding when sick |
| 48 | Pre-lacteals given |
| 49 | Recommended time for initiation of Breastfeeding |
| 50 | Food recommended for children <6 months |
| 51 | Recommended duration of EBF |
| 52 | Benefit of EBF to child |
| 53 | Benefit of EBF to mother |
| 54 | Confident about EBF |
| 55 | Confident to Express |
| 56 | Exclusive breastfeeding for 6 months is good |
| 57 | It’s difficult to exclusively breastfeed for 6 months |
